# Defective Viral Particles Produced in Mast Cells Can Effectively Fight Against Lethal Influenza A Virus

**DOI:** 10.3389/fmicb.2020.553274

**Published:** 2020-11-04

**Authors:** Caiyun Huo, Jinlong Cheng, Jin Xiao, Mingyong Chen, Shumei Zou, Haiyan Tian, Ming Wang, Lunquan Sun, Zhihui Hao, Yanxin Hu

**Affiliations:** ^1^Key Laboratory of Animal Epidemiology of the Ministry of Agriculture, College of Veterinary Medicine, China Agricultural University, Beijing, China; ^2^Key Laboratory of Veterinary Bioproduction and Chemical Medicine of the Ministry of Agriculture, Zhongmu Institutes of China Animal Husbandry Industry Co., Ltd., Beijing, China; ^3^National Institute for Viral Disease Control and Prevention, Collaboration Innovation Center for Diagnosis and Treatment of Infectious Diseases, Chinese Center for Disease Control and Prevention, Key Laboratory for Medical Virology, National Health and Family Planning Commission, Beijing, China; ^4^Center for Molecular Medicine, Xiangya Hospital, Central South University, Changsha, China

**Keywords:** highly pathogenic H5N1 avian influenza virus, mast cells, defective viral particles, therapeutic protection, interferon signaling pathway

## Abstract

Mast cells play an important role in the pathogenesis of highly pathogenic H5N1 avian influenza virus (H5N1-HPAIV) infection. Defective viral particles (DPs) can interfere with the replication of infectious viruses and stimulate the innate immune response of host cells. However, DPs arising from mast cells during HPAIV replication and their potent antiviral actions has not been reported. Here, we showed that the human mastocytoma cell line, HMC-1, allowed for the productive replication of the H5N1-HPAIV. Compared with alveolar cell line A549, DPs were propagated preferentially and abundantly in mast cells following IAV infection, which can be attributed to the wide existence of Argonaute 2 (AGO2) in HMC-1 cells. In addition, DPs generated in H5N1-infected cells could provide great therapeutic protection on mice to fight against various influenza A viruses, which included not only homologous H5N1-HPAIV, but also heterologous H1N1, H3N2, H7N2, and H9N2. Importantly, DPs generated in H5N1-infected HMC-1 cells could diminish viral virulence *in vivo* and *in vitro* by triggering a robust antiviral response through type II interferon signaling pathways. This study is the first to illustrate the arising of DPs in H5N1-HPAIV infected mast cells and explore their favorable ability to protect mice from influenza A viruses infection, which provides a novel insight and valuable information for the progress of new strategies to fight influenza A viruses infection, especially highly pathogenic avian influenza virus infection by focusing on the DPs generated in mast cells.

## Introduction

Influenza A virus (IAV) is highly infectious, and causes significant morbidity and mortality worldwide (Ly et al., 2016; Marinova-Petkova et al., 2016; Thomas et al., 2017). Highly pathogenic avian influenza virus (H5N1-HPAIV) has been categorized as a List A disease by the International Office of Epizootics (OIE). Series of outbreaks raise the perceived public health significance of influenza to a new level. The extensive using of antiviral drugs such as oseltamivir and amantadine has led to drug-resistant strains of influenza emerging. There is an urgent need to develop new approaches to combat HPAIV infection. The excessive pro-inflammatory cytokine production, is the major cause for the lethal clinical symptoms and even high mortality (Cheung et al., 2002; Cilloniz et al., 2009; Cheng et al., 2011). Mast cells are highly granulated cells mainly enriched in areas such as the skin, airway, and digestive tract. Mast cells are pivotal not only in anaphylaxis, but also in the host’s defense against parasites, bacteria and viruses (King et al., 2000; Jolly et al., 2004; Marshall, 2004; Brown et al., 2009; Sun et al., 2009; Meng et al., 2016). Most importantly, we previously demonstrated that mast cells were activated in H5N1-HPAIV infected mice and escalated lung inflammatory injury (Hu et al., 2012; Meng et al., 2016).

Defective viral genomes (DVGs) are truncated viral genomes that are produced at the peak of full-length virus replication (Lazzarini et al., 1981; van den Hoogen et al., 2014; Sun et al., 2015). In the late 1940s, Von Magnus was the first to identify that the influenza virus contained DVGs were also called defective particles (DPs) (Von Magnus, 1951). In recent years, studies have found DPs in PR8-H1N1-infected mice, and in the upper respiratory tissues of patients (Saira et al., 2013; Tapia et al., 2013). DPs lose infectivity due to at least one truncated gene segment (Mercado-Lopez et al., 2013). DVGs mostly occur in PB2, PB1, PA, HA, and M gene segments during IAV infection (Laske et al., 2016; Xue et al., 2016). In addition, DPs are found in various IAV-infected cells, such as human lung adenocarcinoma cell line A549 (Frensing et al., 2014; Xue et al., 2016). DPs can directly interfere with standard virus replication due to their truncated genomic structure and efficient promoter regions (Mercado-Lopez et al., 2013; Tapia et al., 2013; Sun et al., 2015; Laske et al., 2016). In addition, DPs strongly induce type I and type III interferon (IFN) expression of cells during the infection *in vitro* (Easton et al., 2011). Nevertheless, the generation of DPs in mast cells during avian influenza virus infection is still unexplored, especially highly pathogenic avian influenza virus.

Argonaute 2 (AGO2) is a kind of “slicer” RNase, which is the key catalytic component of cleavage-competent RNA-induced silencing complex (RISC) (Hannon, 2002; Van Stry et al., 2012). The AGO2 has been revealed to have the four core domains including N domain, PAZ domain, MID domain, and PIWI domain, which PIWI domain provides catalytic activity to RISC (Ye et al., 2015). Among four of AGO family proteins (AGO1, AGO2, AGO3, and AGO4), only AGO2 exhibits the RNA cleavage (or slicer) and catalytic activity required for small interfering RNA (siRNA) (Liu et al., 2004). Due to its essential role of silencing activity, the cells that are abundant of AGO2 are widely used as the transfection model of RNA interference (RNAi) experiments to silence the target expression of the cognate gene (Barik, 2010). In terms of IAV infection, reports have shown that virus-derived siRNAs were highly produced in infected A549 cells, in which AGO2 plays a crucial role in cleavage and catalysis (Tsai et al., 2018). In addition, AGO2 catalytic activity restricts multiple virus infections in mature cells (Li et al., 2016). The absence of a functional AGO2 protein supports a significantly higher level of replication of wild-type IAV, and also does not give rise to detectable levels of viral siRNAs in infected A549 cells (Tsai et al., 2018). To date, few studies have shown the existence and functions of AGO2 in mast cells.

In the present study, we demonstrated that the HMC-1 cells support the replication of H5N1-HPAIV. We also assessed mast cells for the presence of DPs following H5N1-HPAIV infection by investigating and analyzing the generation, gene segment origin, genome sequence, and function of the DPs induced by IAVs.

## Materials and Methods

### Viruses and Cell Culture

The H5N1 (A/Chicken/Henan/1/04), H1N1 (A/WSN/33), the H3N2 (A/Duck/Anhui/3/14), H9N2 (A/Chicken/Hebei/4/08), and H7N2 (A/Chicken/Hebei/2/02) viruses were isolated and virus titers were determined as described previously (Huo et al., 2018b). The stocks used for originally infection had been purified with low content of DVGs (LD virus). All experiments with the H5N1 virus were conducted in a biosafety level 3 (BSL-3) containment laboratory approved by the Ministry of Agriculture of China.

The human mastocytoma cell line HMC-1 was provided by Medical College of China Three Gorges University. The human lung adenocarcinoma cell line A549, the human acute monocytic leukemia cell line THP-1, the murine macrophages cell line RAW 264.7, the mouse mastocytoma cell line P815, and the Madin-Darby canine kidney cells MDCK were provided by the Cell Resource Center of Peking Union Medical College (Beijing, China) and cultured as described previously (Xie et al., 2014).

### Immunofluorescence Staining and Confocal Microscopy

The expression pattern of SA receptors was performed by immunofluorescence staining and confocal microscopy as described previously (Meng et al., 2016). Cultured cells were fixed on a polylysine-coated slide with 4% formaldehyde, and blocked with 3% BSA. The non-cancerous lung tissue was collected from the normal lung tissue of the patient in Xiangya Hospital of Central South University. Tissue samples were fixed in 4% neutral formalin, embedded in paraffin, and serially cut at a thickness of 5 μm. To visualize surface receptors, slides containing fixed tissues or cells were directly stained with fluorescein- *Sambucus nigra* bark lectin (SNA, specific to SAα2,6-Gal) or fluorescein- *Maackia amurensis* lectin I (MAA-I, specific to SAα2,3-Galβ(1-4)GlcNAc). To detect tryptase expression, cells were permeabilized with 0.5% Triton X-100 before blocking, then tissue sections or cell slides were either incubated with a mouse or rabbit anti-mast cell tryptase monoclonal antibody (Abcam, Hong Kong, China) for 2 h at room temperature. After washing three times with PBS-T, tissue sections were further incubated with a Texas red-conjugated goat anti-mouse or rabbit secondary antibody, and cell slides were incubated with a FITC-conjugated goat anti-mouse secondary antibody (Abcam) for 1 h at room temperature. To visualize the nuclei, all slides were stained with 3 μg/ml 4′,6′-Diamidine-2-phenylindole (DAPI) (Sigma-Aldrich) for 5 min at room temperature and then examined under a laser scanning confocal microscope (Leica TCS SP5 II, Leica Microsystems, Wetzlar, Germany).

### Viral Infection, Transmission Electron Microscopy (TEM) and Plaque Assays *in vitro*

The methods were described previously (Liu et al., 2014; Meng et al., 2016). Briefly, cell monolayers were formed in tissue culture plates by seeding 6-well (1 × 10^6^ cells/well) plates, washed with DMEM and infected with viruses at a multiplicity of infection (MOI) of 10 for 1 h at 37°C. After incubation, cells monolayers were washed and DMEM supplemented with 1% bovine serum albumin was added to each well and incubated for the indicated times. For TEM assay, cells were infected with IAVs at MOI = 10 for 12 h, trypsinized and fixed using 2.5% (v/v) glutaraldehydein PBS for 2 h at 4°C, then the samples were sent to electron microscope laboratory for the preparation of thin section and observation of viral particles in China Agricultural University. For plaque assays, MDCK monolayer cells at 90% confluence in 6-well plates were washed with DMEM and infected with 10-fold serially diluted virus inoculum. After 1 h of incubation at 37°C, the inoculum was removed and washed. Then cell monolayers were overlaid with semisolid agar containing 0.5 mg/ml trypsin tosylsulfonyl phenylalanyl chloromethyl ketone (TPCK) (Sigma-Aldrich). Plaques were fully developed after 72 h at 37°C and 5% CO2. They were then fixed and stained with 1% crystal violet. The plaques were counted and the concentration of the initial viral suspension in PFU (plaque-forming unit)/ml was calculated.

### Real-Time Quantitative PCR (RT-qPCR) and Enzyme Linked Immunosorbent Assay (ELISA)

The gene mRNA levels were determined by RT-qPCR and protein expression was performed by ELISAs, as described previously (Jin et al., 2005; Huo et al., 2017, 2018a). The primer sequences were listed in Supplementary Material (Supplementary Table S1).

### Viral Genome Detection and Nucleotide Sequences of DVGs

One-step PCR and infectivity titer (I)/total titer (T) ratio calculation for viral genome detection were performed as described previously (Xue et al., 2016). The primer sequences were listed in Supplementary Material (Supplementary Table S1). PCR products of the expected length were purified with a Gel Extraction kit (OMEGA, United States) and then cloned into the pEASY-Blunt Cloning Vector (TaKaRa, Japan) according to the manufacturer’s instructions. Sequencing of the cloned genomic fragments was performed by Qingke Company (Beijing, China). The sequences obtained in this study were compared with other IAV sequences available in the GenBank database.

### Virus With Content of DVGs (HD Virus) Isolation and DPs Preparation

For HMC-1 cells, the cells were infected with H5N1 and H1N1 at an equal MOI of 10 for 24 and 30 h, respectively. For A549 cells, the cells were infected with H5N1 at the MOI of 10 for 12 h. For MDCK cells, the cells were also infected with H5N1 at the MOI of 10 for 12 h. Then, the cell supernatants were collected, purified by differential centrifugation (3000r 30 min, 10000r 30 min) and the DPs were detected. These samples were called the HD virus that contained the high numbers of DPs. The helper virus infectivity of HD virus was eliminated by irradiation with UV light for 2 min at 253.7 nm. This had few effect on defective interfering RNA due to the smaller UV-target size of the DVGs (395 nucleotides) than the infectious viral genome (13,600 nucleotides).

### Viral Infection *in vivo* and Histopathological Changes

The procedures of viral infection *in vivo* and detection of histopathological changes by hematoxylin and eosin (H&E) staining and immunohistochemical (IHC) staining were reported previously (Huo et al., 2017). Anti-IAV nucleoprotein (NP) mAb (AA5H, Abcam, Cambridge, MA, United States) were used in IHC staining and were diluted at 1:1000. Female BALB/c mice (8–10 weeks) were purchased from Vital River Laboratories (Beijing, China), and feed pathogen-free food and water in independent ventilated cages. Mice were first anesthetized with Zotile^®^ (Virbac, Carros, France), and then infected intranasally with PBS-diluted virus or DMEM alone. The lung tissues were then collected at day 3 and day 6 post-infection, respectively. Animal experiments were approved by the Animal Ethics Committee of China Agricultural University (approval number 201206078) and were performed according to Regulations of Experimental Animals of Beijing Authority. Besides, experimental protocols conformed to the guidelines of the Beijing Laboratory Animal Welfare and Ethics Committee and were approved by the Beijing Association for Science and Technology (approval number SYXK-2009-0423).

### siRNA Transfection and Viral Infection in Transfected Cells

The procedures were described previously (Zhang et al., 2019). The optimum target sequences used for gene silencing were listed in Supplementary Material (Supplementary Table S2). For transfection, 1 × 10^6^ cells were plated into 6-well plates and incubated overnight. A 5 μL Lipofectamine 2000 reagent (Invitrogen) was diluted in 245 μL Dulbecco’s modified Eagle’s medium (DMEM) and incubated at room temperature for 5 min. Additionally, 100 pmol siRNA duplex or negative control siRNA was diluted in 250 μL DMEM and mixed with pre-diluted Lipofectamine 2000 for 20 min at room temperature, then was added into matching well, respectively. The transfection efficiency was measured by RT-qPCR and western blotting, as reported previously (Zhang et al., 2019). The AGO2 (ab156870), IFNAR1 (sc-7391) and IFNGR1 (sc-28363) antibodies were used according to the instruction manual.

### Data Analysis

Data analysis was conducted using two-way analysis of variance with GraphPad Prism (ver. 5.0). *P* < 0.05 represents statistical significance. The results were showed as the means ± standard deviations of three independent experiments.

## Results

### DPs Were Vastly Propagated in HMC-1 Cells During H5N1-HPAIV Infection

**FIGURE 1:**
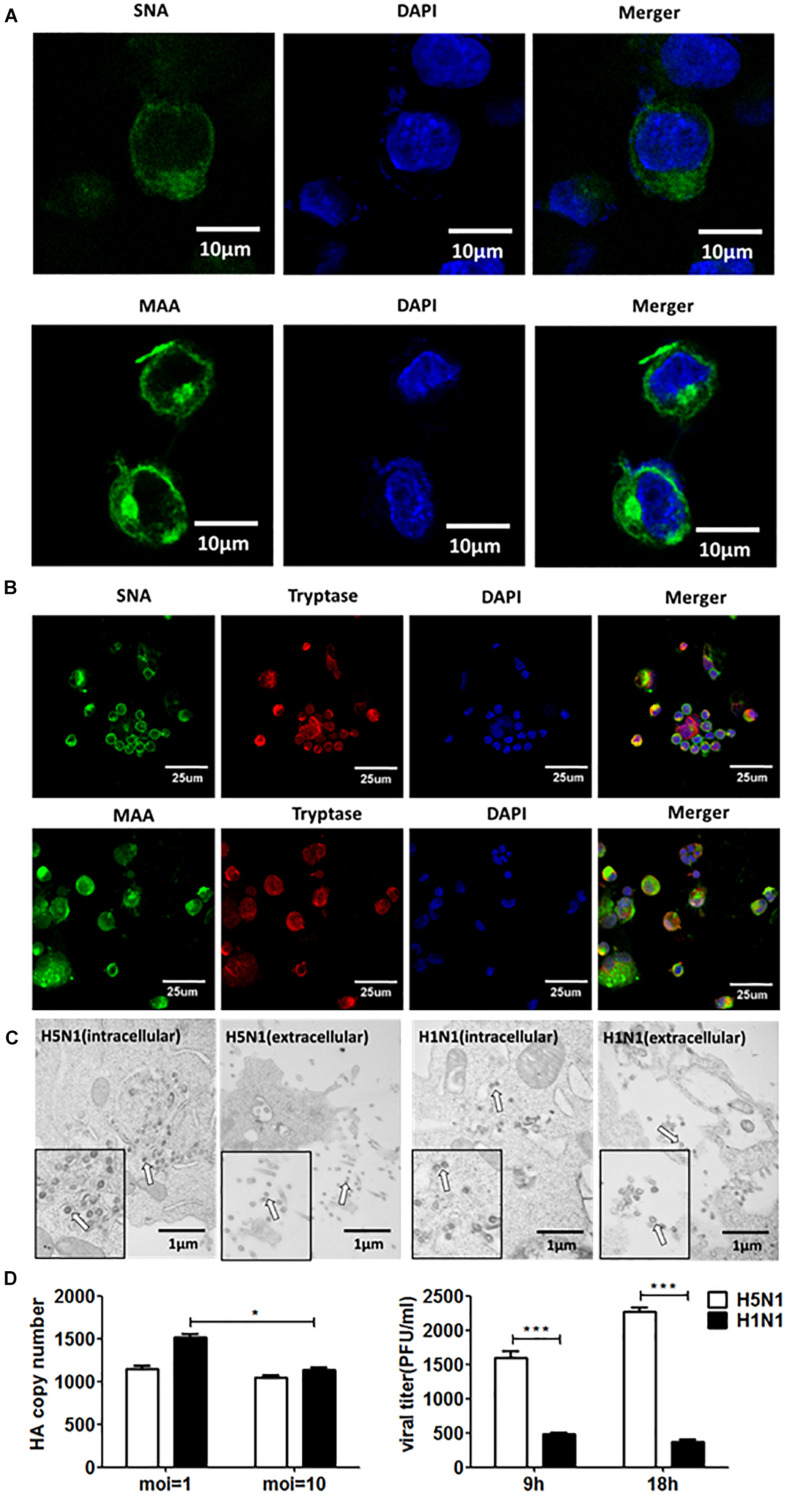
Human mast cells express SA receptors and support productive replication of H5N1-HPAIV. **(A)** and **(B)** HMC-1 cells and representative human lung sections were stained and analyzed by immunofluorescence staining (Green = SA receptors, blue = nuclei and red = tryptase). **(C)** HMC-1 cells were infected with H5N1 and H1N1 at an equal MOI of 10 for 12 h. The viruses located intracellularly or extracellularly were observed using transmission electron microscopy. Arrows denote virus particles. **(D)** HMC-1 cells were infected with H5N1 and H1N1 at an equal MOI of 10 and the viral HA gene expression was quantified by RT-qPCR. The viral titers of culture supernatant were determined using plaque assays. The results presented are from three independent replicates (**P < 0.01; ***P < 0.001; ns, not significant).

Firstly, we detected the replications of H5N1-HPAIV s in HMC-1 cells. Due to that SA receptors are pivotal for the entry of influenza viruses into cells. Here, both α-2,3-linked and α-2,6-linked SA receptors were detected on the surface of HMC-1 cells (Figure 1A). Then, the human lung primary mast cells were also used for further validation of SA receptors and the results were consistent with that of mast cell line (Figure 1B). Positive tryptase staining (red) was used for further identification of the primary mast cells. These results validate that α-2,3- and α-2,6-linked SA receptors are all expressed on the surface of human mast cells. To evaluate if IAVs can replicate productively in HMC-1 cells, we detected the replication kinetics of IAVs in HMC-1 cells by TEM. As shown in Figure 1C, virions could be found in cells following both H1N1 and H5N1 infection at high multiplicity of infection (MOI = 10). On the surface of HMC-1 cells, however, fewer viral particles were present on the surface of H1N1-infected cells compared with H5N1-infected cells. Besides, viral gene expression and plaque formation were also used to further assess the IAV replication in HMC-1 cells. As shown in Figure 1D, viral HA gene could be detected in infected HMC-1 cells. Moreover, the replication of H5N1 virus was relatively lower than that of H1N1 virus. However, plaque assays showed that the numbers of infectious viral particles were higher in H5N1-infected HMC-1 cells compared with H1N1-infected HMC-1 cells, especially at 18 h post infection. Taken together, the results suggested that H5N1-HPAIV could productively replicate in HMC-1 cells.

**FIGURE 2:**
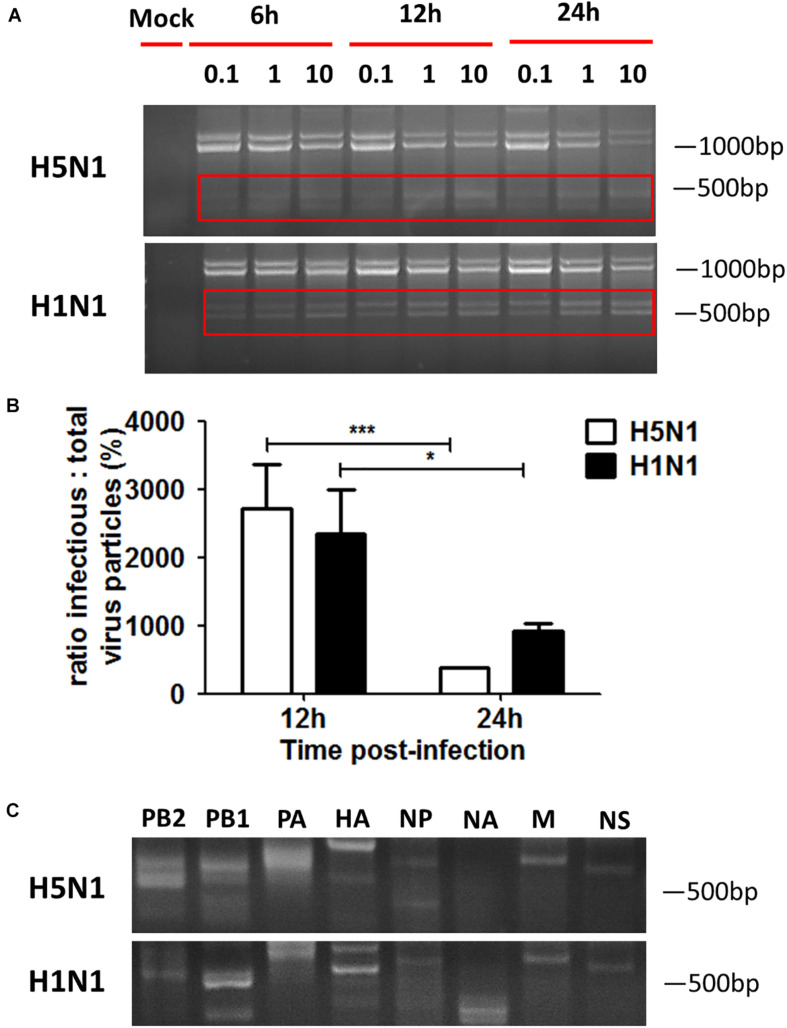
DPs accumulate quickly and abundantly in H5N1 infected HMC-1 cells. HMC-1 cells were infected with H1N1 and H5N1 at an equal MOI of 10 for the periods specified, respectively. **(A)** The viral PCR products were amplified using primers universal for IAV genomic segments. Red/white solid boxes indicate DVGs. **(B)** The infectivity titers (I) of the culture media supernatants were measured using TCID50 assays and total titers (T) were measured using hemagglutination assays. The I/T ratios equaled to TCID50 per 25 μl/HA per 25 μl. The results presented are from three independent replicates (*P < 0.05; ***P < 0.001; ns, not significant). **(C)** The viral PCR products were amplified using primers specific for each IAV genomic segment.

Influenza virus propagation in various cells can favor the propagation of DPs, since the generation of DPs is cell-dependent. Here, we detected if DPs were generated in HMC-1 cells following HPAIV infection. As shown in Figure 2A, DPs that contained the DVGs in length of 300–700 bp were propagated at 6 h post infection and became more abundant over time. At the same infectious time, high MOI could also lead to the increasing numbers of DPs. RT-PCR can only provide a visual quality control, but quantitative measure of the DPs can be achieved by calculating the ratio which is equal to infectious particles (I)/total viral particles (T). Figure 2B showed that the I/T ratio was dramatically decreased in HMC-1 cells at 24 h post infection compared than at 12 h post infection. Besides, H5N1 group had significantly lower I/T ratio than did the H1N1 group at 24 h post infection. We also detected and compared the distinct DVGs in all eight influenza virus genome segments using segment-specific RT-PCR. Eight primers that were specific to each genome segment were used separately to amplify defective interfering RNAs. As shown in Figure 2C, the H5N1 group had more plentiful DVG RNAs from PB2, PB1, and PA segments than the H1N1 group. Furthermore, we tested nucleotide sequences of DVGs in HMC-1 cells and randomly selected nucleotide sequences of these DVGs that occured in PB2 gene fragments were shown in Supplementary Figure S1. We found that DVGs mainly belonged to the deleted DVGs rather than copy-back DVGs. Besides, the sequences of these deleted DVGs were lost randomly and exhibited variety. Taken together, these results demonstrated that DVGs were propagated quickly and abundantly in H5N1-infected HMC-1 cells.

**FIGURE 3:**
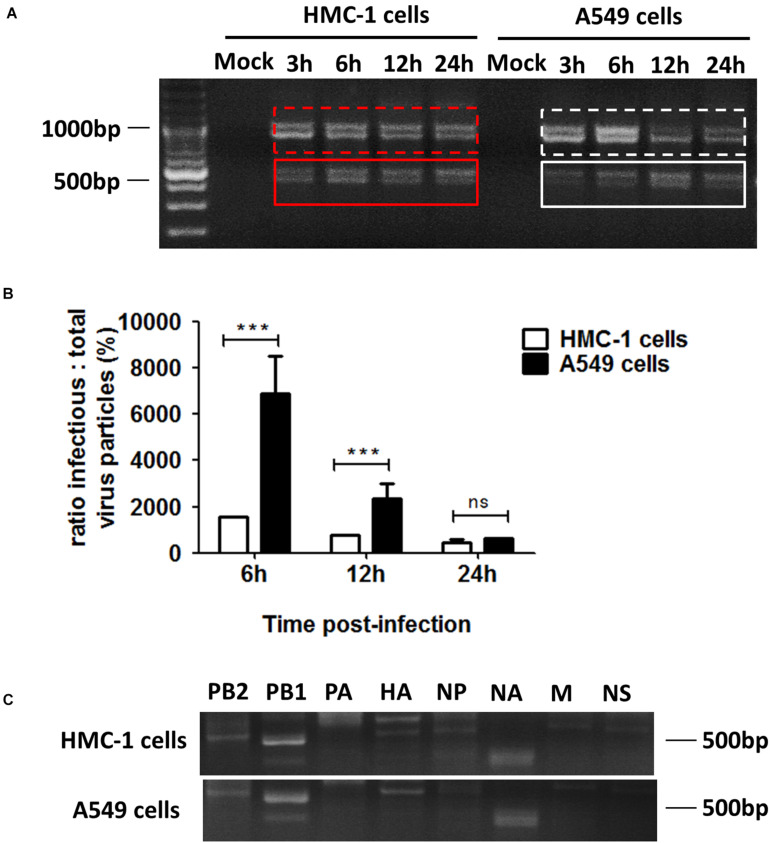
DPs were propagated preferentially and abundantly in HMC-1 cells compared with A549 cells at the early stage of IAV infection. HMC-1 cells and A549 cells were infected with H1N1 at an equal MOI of 1 for the periods specified, respectively. **(A)** The viral PCR products were amplified using primers universal for IAV genomic segments. Red/white solid boxes indicate DVGs. **(B)** The infectivity titers (I) of the culture media supernatants were measured using TCID50 assays and total titers (T) were measured using hemagglutination assays. The I/T ratios equaled to TCID50 per 25 μl/HA per 25 μl. The results presented are from three independent replicates (***P < 0.001; ns = no significance). **(C)** The viral PCR products were amplified using primers specific for each IAV genomic segment.

Considering that cell types also have an influence on the generation of DPs, we thus investigated the differences in DPs between immune cells and non-immune cells following IAVs infection. DPs are known to be propagated in H1N1 infected A549 cells, so we selected the A549 cells to be the non-immune cell model. HMC-1 and A549 cells were infected with H1N1 and the cells and supernatants were harvested. For HMC-1 cells, DVGs could be present at 3 h after H1N1 infection and became more abundant over time. However, weak DVGs were detected in A549 cells from 6 h after H1N1 infection (Figure 3A). Since there were no visually obvious differences in DVGs at 12 or 24 h, I/T ratios were also measured. As shown in Figure 3B, the I/T ratios in HMC-1 cells were significantly lower than those in A549 cells at both 6 and 12 h after H1N1 infection. Segment-specific RT-PCR also showed some difference of DVGs between the two cells at 6 h after H1N1 infection (Figure 3C). Consistent with the above data, these results demonstrated that DPs were produced more preferentially and abundantly in HMC-1 cells than in A549 cells at the early stage of IAV infection.

### High AGO2 Expression Was Associated With DPs Propagation and Restriction of Wildtype Virus Replication During IAV Infection in HMC-1 Cells

**FIGURE 4:**
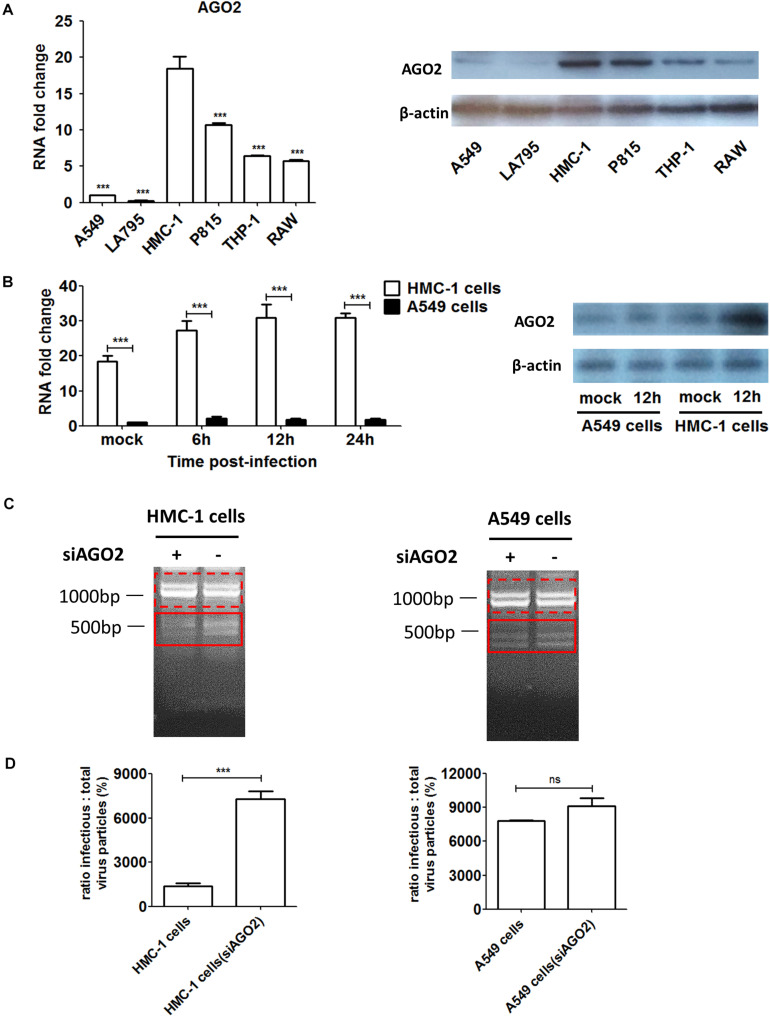
The AGO2 is widely abundant in HMC-1 cells and plays an essential role in DPs propagation and restriction of wildtype virus replication during IAV infection. **(A)** The gene expression and protein of AGO2 in non-infected HMC-1cells, A549 cells, LA795 cells, P815 cells, THP-1 cells, and RAW cells were detected by RT-qPCR and western blotting, respectively. **(B)** HMC-1 cells and A549 cells were infected with H1N1 at an equal MOI of 1 for the periods specified, respectively. Cells were collected. The gene expression and protein expression of AGO2 in infected- HMC-1 cells and A549 cells were detected by RT-qPCR and western blotting, respectively. **(C)** HMC-1 cells and A549 cells were transfected with siAGO2 and then were infected with H1N1 for 6 h. The viral PCR products were amplified using primers universal for IAV genomic segments. Red dotted boxes indicate IAV genomes. Red solid boxes indicate DVGs. **(D)** HMC-1 cells and A549 cells were transfected with siAGO2 and then were infected with H1N1 for 6 h. The I/T ratios equaled to TCID50 per 25 μl/HA per 25 μl. The results presented are from three independent replicates (ns, not significant; ***P < 0.001).

To investigate whether AGO2 was present in immune cells, we detected and compared the expression levels of AGO2 in different cells. As shown in Figure 4A, both RNA and protein levels of AGO2 were significantly higher in immune cells than that in epithelial cells. Among four types of immune cells, mast cells expressed much more AGO2 than THP-1 or RAW cells did. In particular, significant enrichment of AGO2 could be seen in HMC-1 cells. Due to the abundance of AGO2 in HMC-1 cells, we further explored whether the IAV infection could affect the amount of AGO2 in infected cells. HMC-1 cells and A549 cells were infected with H1N1 virus, respectively. The cells were harvested at 6, 12, and 24 h after H1N1 infection and AGO2 gene expression was measured by qPCR and western blotting (Figure 4B). During IAV infection, mRNA expression and protein expression of AGO2 were all dramatically increased in the infected-HMC-1 cells than in non-infected HMC-1 cells. However, IAV infection could not affect the mRNA expression of AGO2 in A549 cells. The above results indicated that the AGO2 were abundant in immune cells (especially in HMC-1 cells) and IAV infection could promote the further production of AGO2 in infected HMC-1 cells, suggesting its potential role in mast cells upon IAV infection.

In order to further investigate the potential contribution of AGO2 to DPs production as well as virus replication during IAV infection, siRNA was used to knockdown the AGO2 in HMC-1 and A549 cells, respectively. As shown in Figure 4C, RT-PCR showed that knocking down AGO2 in HMC-1 cells could obviously weaken the DVGs expression at 6 h after H1N1 infection, while no visually obvious differences were seen in A549 cells (siAGO2) group. To further compare the effects of AGO2 on DPs content, I/T ratios were also measured. As shown in Figure 4D, knocking down AGO2 could increase the I/T ratios in both cells, indicating that the loss of AGO2 could cause the reduced DPs in infected cells. Notably, the DPs were dramatically decreased in AGO2 knockdown HMC-1 cells, while no significant reduction was shown in AGO2 knockdown A549 cells. Together, these data further indicated that the AGO2 was abundant in HMC-1 cells and played an essential role in DPs propagation and restriction of wildtype virus replication during IAV infection.

### DPs Generated in H5N1 Infected Mast Cells Have a Great Therapeutic Effect on Mice to Fight Against HPAIV and Other IAVs

**FIGURE 5:**
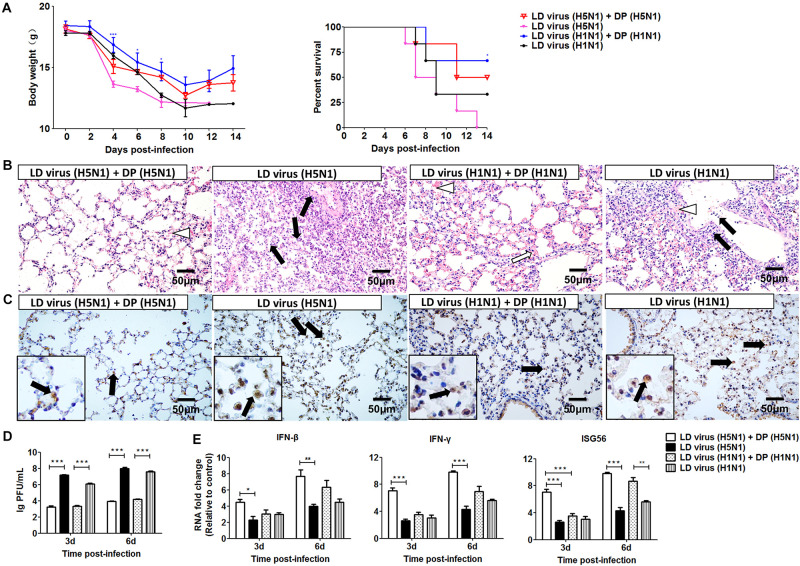
DPs generated in H5N1 HPAIV infected mast cells protect mice from homologous influenza viruses. Mice were challenged with 100 TCID50/mouse LD virus alone or in the presence of 25 μl/mouse purified DPs. Mice received DP (H5N1), DP (H1N1), or DMEM immediately following homologous virus challenge (*N* = 6). Statistical difference was analyzed by comparing each group with LD virus (H5N1) group (*P < 0.05; ***P < 0.001). **(A)** Weight loss and survival rates were recorded during infection. **(B)** Lung pathological changes were observed by H&E staining. Black arrows indicate lymphocytic infiltration. Hollow arrows indicate hyperemia. Hollow triangles indicate hemorrhage. **(C)** The expression of viral NP was measured using IHC staining. Black arrows indicate positive signals. **(D)** The viral titers were measured using plaque assay. **(E)** The expression of IFN-β, IFN-γ, and ISG56 in lung was measured by RT-qPCR. Graphs are shown from three independent replicates (*P < 0.05; **P < 0.01; ***P < 0.001).

To investigate whether the DVGs generated in infected HMC-1 cells could provide homologous protection against the serious disease caused by simultaneous lethal IAVs infection, mice were infected with 100 TCID50/mouse H5N1 or H1N1 LD virus and received homologous DP or DMEM control immediately following virus inoculation. Then, the HD virus was treated by UV irradiation, which could make HD virus lose activity while retaining DVG RNAs of DPs. HD viruses were isolated and prepared from H5N1-infected and H1N1-infected HMC-1 cells, respectively. As shown in Table 1, H5N1 virus stocks and H1N1 virus stocks, which maintained a low DVG content, were regarded as LD virus. HD viruses isolated from H5N1-infected and H1N1-infected HMC-1 cells had the same I/T ratios, which were much lower than found in LD viruses. Then, the infectivity detection of prepared defective particles was performed and the results showed that these DPs had no negative effect on body weight *in vivo* (Supplementary Figure S2). Figure 5A showed that DP (H5N1) or DP (H1N1) could protect mice from the weight loss and morbidity caused by homologous LD virus. Besides, histopathological changes in the lungs were also examined by H&E staining (Figure 5B). As expected, the lungs of mice in the LD virus groups had severe lesions, including hyperemia, structural damage of bronchial and alveolar as well as significant lymphocyte and inflammatory cell infiltration surrounded the blood vessels and bronchioles. No obviously histopathologic changes were seen in the LD virus (H5N1) + DP (H5N1) group and LD virus (H1N1) + DP (H1N1) group, and only mild hyperemia was present in the alveoli. As shown in Figure 5C, the IHC staining results by using an IAV nucleoprotein (NP) antibody also showed fewer positive cells in lung of mice in the LD virus (H5N1) + DP (H5N1) group and LD virus (H1N1) + DP (H1N1) group. The viral titers were also downregulated dramatically in these two groups (Figure 5D). To examine the antiviral response induced by DPs, we used RT-qPCR to measure kinetic profiles for IFN-β, IFN-γ, and ISG56, which are potentially involved in antiviral responses. Figure 5E showed that the gene expression of the cytokines was augmented in the LD virus (H5N1) + DP (H5N1) group and LD virus (H1N1) + DP (H1N1) group, especially at day 6 post-infection. Interestingly, LD virus (H5N1) + DP (H5N1) group showed the highest expression levels of antiviral cytokines among these groups, indicating the strongest antiviral responses. These results could confirm the critical role of DP (H5N1) and DP (H1N1) in protecting mice from the serious disease caused by simultaneous challenge with lethal IAVs infection.

**TABLE 1:** I/T ratios in HD virus isolated from HMC-1 cells following IAVs infection.

	Time of sample collection	Infectivity titer (I): TCID50/25 μl	Total titer (T): HA/25 μl	I/T
HD virus (H5N1)	24 h	6264.84	16	391.6
LD virus (H5N1)	/	9929102.93	256	3.9 × 10^4^
HD virus (H1N1)	30 h	6264.84	16	391.6
LD virus (H1N1)	/	9929102.93	256	3.9 × 10^4^

**FIGURE 6:**
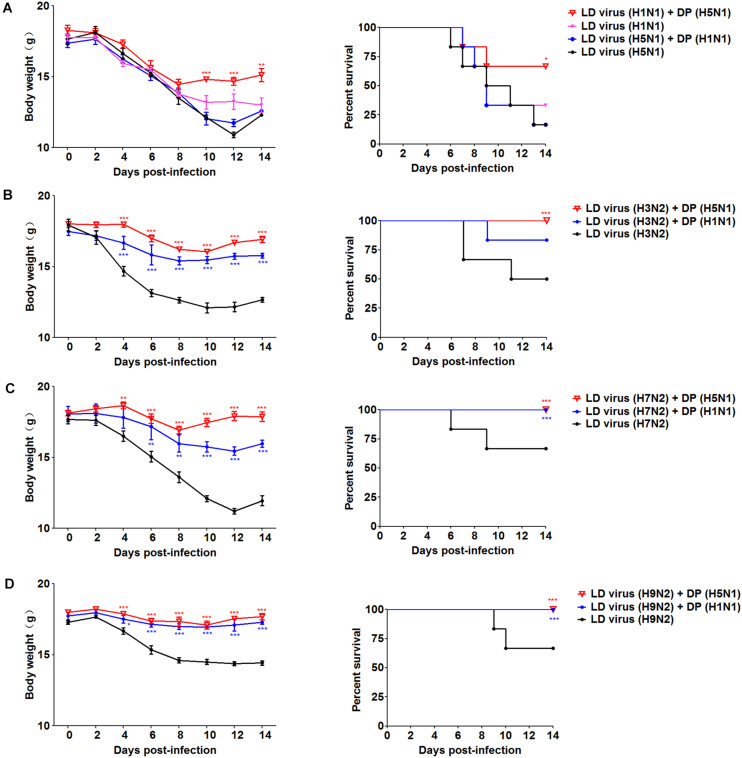
DPs generated in H5N1 HPAIV infected mast cells protect mice from heterologous influenza viruses. Mice were challenged with **(A)** 100 TCID50/mouse LD virus (H1N1/H5N1) or **(B–D)** 10^6^ TCID50/mouse LD virus (H3N2/H7N2/H9N2), alone. Mice received DP or DMEM immediately following heterologous virus inoculation (*N* = 6). Weight loss and survival rates were recorded during infection. Statistical difference was analyzed by comparing each group with LD virus (H5N1) group, LD virus (H3N2) group, LD virus (H7N2) group, and LD virus (H9N2) group, respectively. *P < 0.05; **P < 0.01; ***P < 0.001.

**FIGURE 7:**
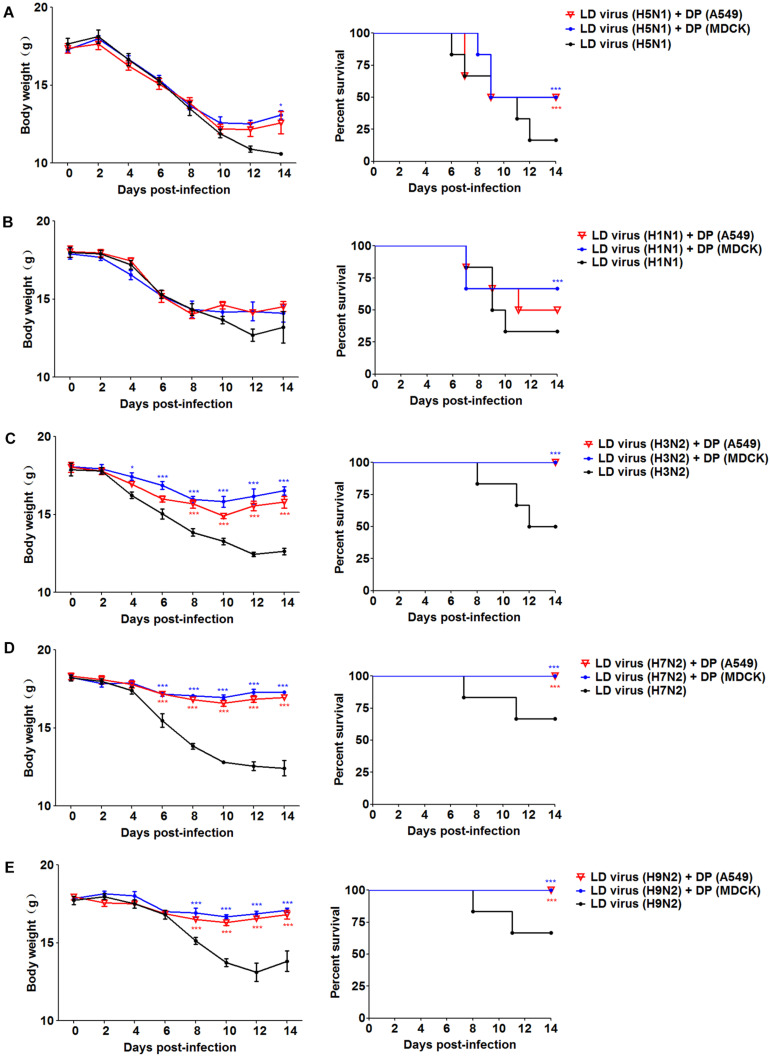
DPs generated in H5N1 HPAIV infected A549 cells and MDCK cells protect mice from various influenza viruses. Mice were challenged with **(A,B)** 100 TCID50/mouse LD virus (H5N1/H1N1) or **(C–E)** 10^6^ TCID50/mouse LD virus (H3N2/H7N2/H9N2), alone. Mice received DP (A549), DP (MDCK), or DMEM immediately following homologous and heterologous virus inoculation, respectively (*N* = 6). Weight loss and survival rates were recorded during infection. Statistical difference was analyzed by comparing each group with LD virus (H5N1) group, LD virus (H1N1) group, LD virus (H3N2) group, LD virus (H7N2) group, and LD virus (H9N2) group, respectively. *P < 0.05; ***P < 0.001.

To explore whether the DPs generated in infected HMC-1 cells protect mice from heterologous virus challenge, the viruses (H3N2, H7N2, and H9N2) that have a low DVG content were also used. As shown in Figure 6, DP (H5N1) could reduce weight loss and increase survival in H1N1, H3N2, H7N2, and H9N2-infected mice compared with LD virus alone. In terms of DP (H1N1), although this kind of DP could fight against H3N2, H7N2, and H9N2, it did not have any protective effect on H5N1 infection. Except for mast cells, we also isolated HD viruses from H5N1-infected A549 cells and MDCK cells that had the same I/T ratios (Table 2). Then, we prepared these two kinds of DPs by irradiation with UV light, and investigated their therapeutic effects on mice to fight against various IAVs. As shown in Figure 7, no serious clinical disease or weight loss was seen after treatment with DP (A549) or DP (MDCK), whereas LD virus caused decreased weight loss and a high mortality rate. Together, the results suggest that the DPs generated in H5N1-infected cells could greatly protect mice from the serious disease caused by HPAIV and other heterologous influenza virus infection.

**TABLE 2:** I/T ratios in HD virus isolated from A549 cells and MDCK cells following H5N1 infection.

	Time of sample collection	Infectivity titer (I): TCID50/25 μl	Total titer (T): HA/25 μl	I/T
HD virus (A549)	12 h	6264.84	16	391.6
HD virus (MDCK)	12 h	6264.84	16	391.6
LD virus	/	9929102.93	256	3.9 × 10^4^

### DPs Generated in H5N1-HPAIV Infected Mast Cells Can Attenuate Viral Virulence *in vivo* and *in vitro* by Triggering a Robust Antiviral Response Through the Type II IFN Signaling Pathways

**FIGURE 8:**
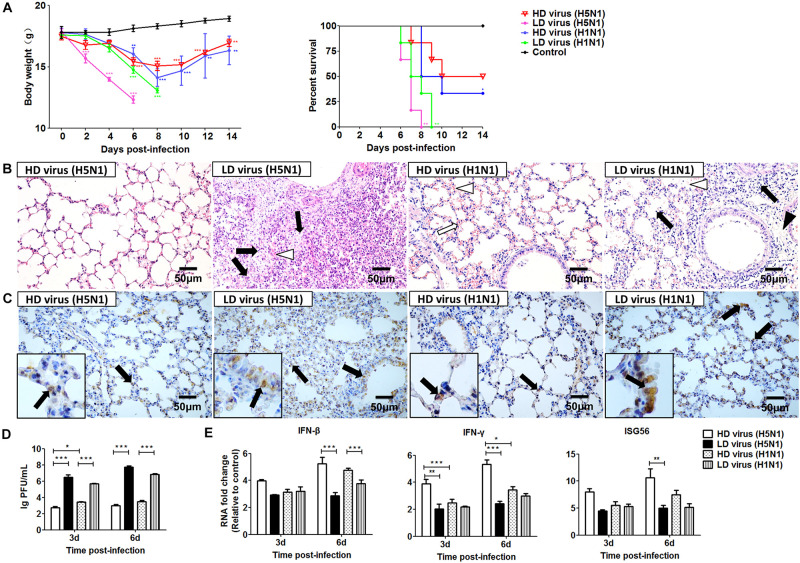
DPs generated in H5N1 HPAIV infected mast cells diminish viral virulence *in vivo*. Mice were infected with 120 TCID50/mouse of HD virus (H5N1), HD virus (H1N1), LD virus (H5N1), or LD virus (H1N1). **(A)** Weight loss and survival rates were recorded during infection. Statistical difference was analyzed by comparing each group with control group. *P < 0.05; **P < 0.01; ***P < 0.001). **(B)** Lung pathological changes were observed by H&E staining. Black arrows indicate lymphocytic infiltration. Hollow arrows indicate hyperemia. Hollow triangles indicate hemorrhage. **(C)** The expression of viral NP was measured using IHC staining. Black arrows indicate positive signals. **(D)** The viral titers were measured using plaque assay. **(E)** The expression of IFN-β, IFN-γ, and ISG56 in lung was measured by RT-qPCR. Graphs are shown from three independent replicates (*P < 0.05; **P < 0.01; ***P < 0.001).

To evaluate the impact of DVGs generated in H5N1-infected cells on viral virulence, the virulence of the HD viruses (H5N1) and HD viruses (H1N1) *in vivo* were investigated. Mice were challenged with the two types of 120 TCID50/mouse HD viruses or LD virus, and animal survival was measured. Figure 8A showed that mice infected with HD virus had a higher mean body weight and reduced morbidity compared with mice challenged with LD virus. Pathological changes in the lungs were examined by H&E staining (Figure 8B). In the HD virus (H5N1) group, no pathological changes were seen and the lung tissue remained an intact structure. Slight changes were detected in the HD virus (H1N1) group, including mild hyperemia in the alveoli. Nevertheless, the two LD virus groups showed the much more severe damage, such as hyperemia, the structural damage of alveolar and bronchial as well as lymphocytes and inflammatory cell infiltration, especially in the LD virus (H5N1) group. Besides, the IHC staining was also taken by using an IAV NP antibody to assess the viral load in the lungs (Figure 8C). Less positive signals were seen in lung epithelial cells from the HD virus (H5N1) group or HD virus (H1N1) group, indicating a lower viral load in mice infected with HD virus. Plaque assay could further validate that HD virus (H5N1) group could decrease the viral titers in lung of infected mice (Figure 8D). Moreover, lung tissue samples were collected to assess the antiviral response by measuring the mRNA expression profiles of IFN-β, IFN-γ, and ISG56 using RT-qPCR (Figure 8E). The production of antiviral cytokines was upregulated notably in the HD virus (H5N1) group compared with the any other groups, especially at day 6 post-infection.

**FIGURE 9:**
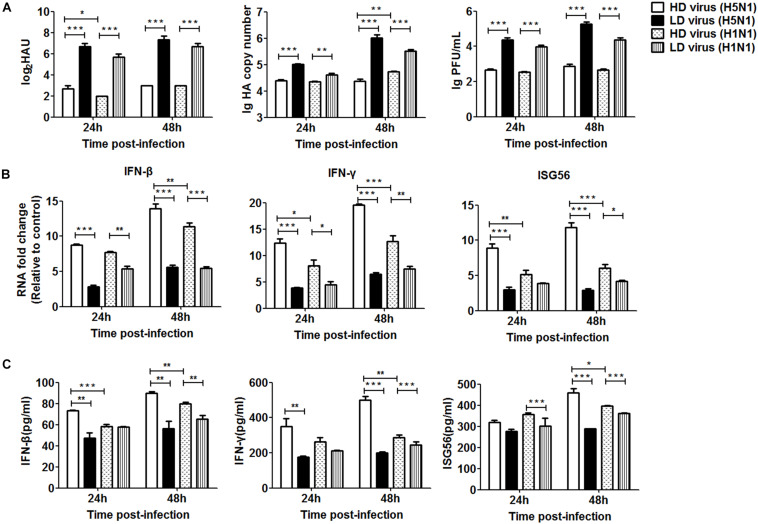
DPs generated in H5N1 HPAIV infected HMC-1 cells diminish viral virulence *in vitro*. HMC-1 cells were infected with H5N1 and H1N1 with low DVG content (LD virus) at an equal MOI of 10, respectively. Culture media supernatant containing an equal and high content of DPs (HD virus) was selected for subsequent experiments. Then, the A549 cells were infected *in vitro* at an equal MOI of 0.2 for the indicated times. **(A)** The viral titers were measured using hemagglutination and plaque assays. The HA gene was analyzed by RT-qPCR. **(B,C)** The expression of IFN-β, IFN-γ, and ISG56 was measured by both RT-qPCR and ELISA. Graphs are shown from three independent replicates (*P < 0.05; **P < 0.01; ***P < 0.001).

To further investigate the differential impact of DVGs generated in H5N1-infected cells on viral virulence, the *in vitro* study was used for validation. As shown in Figure 9A, the viral titers and HA mRNA levels were decreased significantly in HD virus groups compared with the LD virus group. Notably, significantly lower levels of HA gene expression were found in the HD virus (H5N1) group than in the HD virus (H1N1) group, especially at 48 h post-infection. To examine the antiviral response induced upon HD virus infection in more detail, we used PCR and ELISA to measure kinetic profiles for IFN-β, IFN-γ, and ISG56, which are potentially involved in antiviral responses. Figures 9B,C showed that the gene expression and secretion of the cytokines was augmented in the HD virus (H5N1) group compared with the other three groups. These *in vitro* results are in accordance with the *in vivo* results described above, suggesting that DVGs generated in H5N1-infected cells could attenuate viral virulence by triggering a robust antiviral response and thus have the potential to increase the survival of infected mice and suppress excessive inflammation.

**FIGURE 10:**
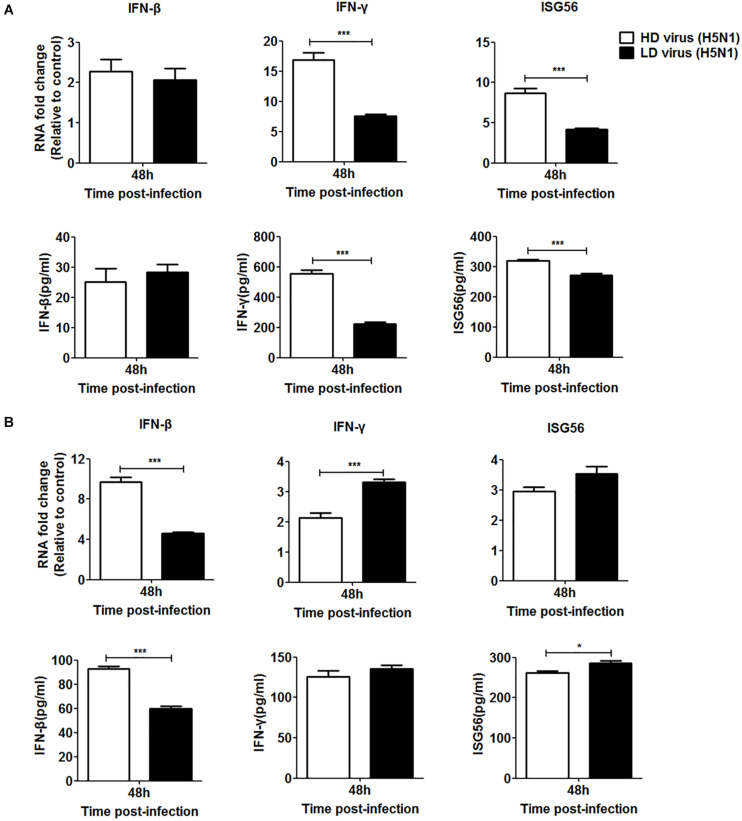
DPs generated in H5N1 HPAIV HMC-1 cells trigger a robust antiviral response *in vitro* via the type II IFN signaling pathways. **(A)** A549 cells were transfected with siIFNAR1 for 36 h and then were infected were infected with HD virus (H5N1) or LD virus. The expression of IFN-β, IFN-γ, and ISG56 was analyzed by RT-qPCR and ELISA. **(B)** A549 cells were transfected with siIFNGR1 for 36 h and then were infected were infected with HD virus (H5N1) or LD virus. The expression of IFN-β, IFN-γ, and ISG56 was measured by RT-qPCR and ELISA. Graphs are shown from three independent replicates (*P < 0.05; ***P < 0.001).

Since the IFN signaling pathway plays a crucial role in the antiviral response during IAV infection, we investigated whether the type I and type II IFN signaling pathways were involved in the antiviral response triggered by the DVGs generated in H5N1-infected cells. siRNA was used to knockdown IFN receptors so that IFN could not bind to them effectively. Specific siRNAs inhibited the expression of IFNAR and IFNGR in a time-dependent manner (Supplementary Figure S2). Since the siRNAs targeting IFNAR or IFNGR had the strongest inhibitory effect at 36 h post-transfection, this transfection time was selected for subsequent experiments. At 36 h post-transfection, cells were infected with HD or LD virus. Knocking down IFNAR reduced mRNA expression and protein secretion of IFN-β in the HD virus group and no difference could be seen between HD virus group and LD virus group, but the expression levels of IFN-γ and ISG56 remained higher in the HD virus group than LD group (Figure 10A). The knock-down of IFNGR dramatically decreased the expression of IFN-γ and ISG56 in the HD virus (H5N1) group and lower than LD virus (H5N1) group (Figure 10B). Combined with the cytokine expression results *in vivo*, we can conclude that the IFN signaling pathway (mainly type II IFN) participates in the antiviral response triggered by the DVGs generated in H5N1-infected cells.

## Discussion

Mast cells, one kind of critical immune cells, are known to have a pivotal role in immunological responses during H5N1 HPAIV infection in our previous study (Hu et al., 2012). Previous research by Marcet et al. (2013) demonstrated that IAV replicated to a limited extent in human mast cells, which indicated the potential antiviral effects of human mast cells against IAV. In accordance with the previous study, we demonstrated that although viral RNA transport, replication as well as viral protein synthesis occurred in human mast cells during H5N1 and H1N1 infection, the infectivity was decreased in mast cells with the extended time. Notably, mouse mast cells were demonstrated to support the productive replication of IAVs *in vitro* at MOI = 0.1 in our previous study (Meng et al., 2016). In our study, we found that human mast cells exhibited very limited replication of any subtypes of IAVs at low dosages such as MOI = 0.1 and MOI = 1, and the replication could only be supported effectively when human mast cells were infected with IAVs at MOI = 10. However, the reason underlying the limited replicative ability of IAVs in human mast cells is poorly understood and needs to be clarified. Here, our results provide the first evidence that human mast cells support effective replication of H5N1 HPAIV.

The first step of IAV infection and viral replication is to recognize and bind to SA receptors, which are widely located on the surface of cells. The main receptor types are α-2,3-linked and α-2,6-linked SA receptors (Matrosovich et al., 2004). In terms of IAVs, human influenza viruses preferentially recognize α-2,6-linked SA receptors, whereas avian influenza viruses mainly recognize α-2,3-linked SA receptors, indicating that the type of SA receptor has a decisive role in influenza virus tropism (Van Poucke et al., 2010; Raman et al., 2014). Here, we confirmed that these two types of SA receptors are all enriched on the surface of human mast cells. To our knowledge, this is the first time that SA receptors are located on the surface of mast cells derived from humans. Besides, the human lung primary mast cells from the lung tissue of the patient were also used in our study for further validation of SA receptors, and the results were consistent with that of mast cell line. Thus, we consider that the observations obtained with a transformed cell line (HMC-1) probably could be extrapolated to a natural infection in animals or human patients.

DPs have two crucial functions: interfering with full-length virus replication and inducing antiviral responses in various cells during IAV infection. Considering the interfering and antiviral activity of DPs, we evaluated the generation and biological role of DPs in human mast cells during H5N1 HPAIV infection. The current study generated novel data and demonstrated for the first time that DPs were propagated quickly and abundantly in H5N1-infected HMC-1 cells at MOI = 10 and provided primary danger signals to trigger the host response.

During IAV infection, the ability to produce DPs during virus growth, and the source gene segments, depend on the virus subtypes. Here, the study offered the first description of distinct in generative ability among the diverse IAVs in human mast cells. We revealed that H5N1 and H1N1 could lead to abundant DPs propagation in HMC-1 cells, especially H5N1. In addition, further comprehensive and detailed analysis of DVGs sources showed that the gene segments of DVGs also differed between H5N1 and H1N1 infection. Previous studies suggested that the functions of DVGs could differ among specific truncated segments. DVGs occurring in PB2, PB1, PA, and/or M gene segments are capable of directly interfering with viral replication, whereas DVGs with the partial deletion of NS1 gene segments mainly induce a type I IFN response in the hosts. Thus, it remains unclear what types of truncated gene segments generated in H5N1-infected mast cells play essential roles and how they function.

Among cases infection by the same virus, cell types have an influence on the ability to produce DPs and its sources of gene segments. Mast cells, which are pivotal in immunity and host defenses, play important roles in both innate and protective immunity against certain viral infections via releasing various cytokines and chemokines. Unlike lung epithelial cells, which are non-immune cells, mast cells are a crucial immune cell type for alerting the immune system to encountered microbes. Previous studies have validated that DPs could be propagated in H1N1 infected A549 cells. Thus, we further investigated the relationship between DPs and cell type. This study was the first to confirm that DPs were propagated preferentially and more abundantly in HMC-1 cells than A549 cells at the early stage of H1N1 infection. As DPs have robust interfering and antiviral activities, we believe that the stronger antiviral responses initiated by mast cells to fight virus infections were tightly associated with the generation of a wide range of DPs. Thus, the results illustrate firstly that the DPs are a critical factor for triggering the stronger antiviral responses in mast cells compared with epithelial cells during IAV infection.

AGO2, one of “slicer” RNase, is a multifunctional protein that belongs to the core catalytic component of RISC. It has various of functions such as cleavage (or slicer) and catalytic activity (Hannon, 2002). The distribution of AGO2 in cells could depend on cell types, and A549 cells have been proved to possess this kind of proteins (Tsai et al., 2018). However, few researches have deeply investigated the existence and functions of AGO2 in immune cells. Here, we provide the novel data that AGO2 is much more abundant in immune cells such as mast cells and macrophages compared to epithelial cells. Interestingly, HMC-1 cells have the larger amount of AGO2 than any other cells, suggesting the crucial role of AGO2 in HMC-1 cells. During IAV infection, AGO2 has also been demonstrated to prompt the production of small interfering RNA and restrict the virus replication (Tsai et al., 2018). In our study, we detected whether the IAV infection affect the AGO2 expression in mast cells and epithelial cells. During IAV infection, the amount of AGO2 was significantly promoted in HMC-1 cells than A549 cells at 12 h and 24 h after H1N1 infection. By coincidence, DPs were also propagated more abundantly in infected HMC-1 cells at the same period time. We speculate that increased AGO2 could facilitate the propagation of DPs in HMC-1 cells to fight against IAV infection. To validate our speculation, the knockdown was taken to make the deficiency of AGO2 in HMC-1 cells and A549 cells. Then, we found that the production of DVGs was dramatically decreased in HMC-1 cells with the transfection of siAGO2 RNA. Thus, we illustrate for the first time that the AGO2 abundant in HMC-1 cells plays an essential role in DPs propagation and restriction of wildtype virus replication during IAV infection.

Since DPs are non-infectious and have a robust ability to activate the host immune response, their clinical antiviral function has attracted significant research interest (Dimmock and Easton, 2015). DPs are always prepared using A549 cells as the *in vitro* model, and antiviral effects are confirmed (Xue et al., 2016). Here, we investigated whether DVGs generated in IAV-infected mast cells also have the desired antiviral potential. Ultraviolet irradiation of HD virus with a high DVGs content for a short period of time (30 s to 2 min) could eliminate full-length virus infectivity while preserving the activity of the remaining DVGs, and thus be used as a method of protecting mice from viral infections. Here, we prepared DPs from H5N1-infected and H1N1-infected cells successfully and compared the protective efficacy of the resulting DPs. DP (H5N1) could protect from H5N1-induced disease more efficiently, promoting the antiviral response and finally increasing the survival of infected mice. Consistent with the H5N1 data, DP (H5N1) also protected against heterologous H1N2, H3N2, H7N2, and H9N2 IAVs *in vivo*. Notably, DP (H1N1) afforded little protection against the serious disease caused by a highly pathogenic lethal H5N1 infection. Thus, these results represent a significant step forward in validating the use of DP (H5N1) as an antiviral method against various IAVs.

Under conditions with the same content of viral titers, the DPs generated in H5N1-infected HMC-1 cells could diminish viral virulence, triggering the robust release of IFNs such as IFN-β and IFN-γ. Regarding the cellular mechanisms of the robust activation of the antiviral response by DPs, previous studies mainly focused on the critical role of the type I signaling pathway. However, the role that the type II signaling pathway plays in the antiviral response was poorly explored. Here, we provide novel data demonstrating that DPs generated in H5N1-infected HMC-1 cells could trigger a robust antiviral response that is dependent on both type I and type II IFN feedback. In addition, knocking down IFNGR dramatically downregulated the expression of immune cytokines in siRNA-transfected cells following HD virus (H5N1) infection, to levels even lower than those that occur following LD virus (H5N1) infection. However, IFNAR knockdown did not cause the same or similar changes in these cytokines. In the absence of IFNAR, which binds to type I IFN, HD virus (H5N1) also increases the expression of IFN-γ and ISG56 compared with LD virus (H5N1). Together, these results confirm that the type II IFN signaling pathway has a much greater role in the antiviral response triggered by the DVGs generated in H5N1-HPAIV infected HMC-1 cells. *Nigel J. Dimmock et al*. demonstrates that cloned DPs, which carry the 244 395nt defective segment 1 RNA and are amplified in MDCK cells, could protect mice from lethal infection with IAVs from several different subtypes and a genetically unrelated respiratory virus. The mechanism of their protection is achieved by stimulating type I IFN and possibly other elements of innate immunity. In our study, we also found that type II IFN signaling pathway had a much greater role in the antiviral response triggered by DPs. Thus, we speculate that the mechanism of cross protection against heterologous virus infection mediated by DPs generated in mast cells is mainly through the function of activating the host immune response.

## Conclusion

In summary, the present data suggest that DPs were propagated quickly and abundantly in mast cells following H5N1 HPAIV infection. Furtherly, DPs effectively generated in mast cells involved with AGO2 abundant existence, which may play an essential role in DPs propagation and restriction of wildtype virus replication during IAV infection. Besides, DPs generated in H5N1-HPAIV infected cells could provide effective therapeutic effect on mice to fight against highly pathogenic H5N1 avian influenza and other various IAVs infection by triggering type II IFN signaling pathways.

## Data Availability Statement

The original contributions presented in the study are included in the article/Supplementary Material, further inquiries can be directed to the corresponding author.

## Ethics Statement

The animal study was reviewed and approved by Animal Ethics Committee of China Agricultural University.

## Author Contributions

CH, JC, LS, and YH carried out experiments, analyzed data, and wrote the manuscript. JX, MC, SZ, HT, MW, LS, ZH, and YH designed the study and supervised the project. CH, JC, LS, and YH assisted in the data analysis and discussion. CH and YH drew the figures. All authors read and approved the final manuscript.

## Conflict of Interest

JX was employed by the company Zhongmu Institutes of China Animal Husbandry Industry Co., Ltd. The remaining authors declare that the research was conducted in the absence of any commercial or financial relationships that could be construed as a potential conflict of interest.
