# Supplementary material for: Defective Viral Particles Produced in Mast Cells Can Effectively Fight Against Lethal Influenza A Virus
Source: Front Microbiol. 2020 Nov 4;11:553274. doi: 10.3389/fmicb.2020.553274 (PMC7671969; doi:10.3389/fmicb.2020.553274)
Supplement: Supplementary file 1 [file Data_Sheet_1.docx]

**Supporting information**

**Defective viral particles produced in mast cells can effectively fight against lethal influenza A virus**

Caiyun Huo^1†^, Jinlong Cheng^1†^, Jin Xiao^2^, Mingyong Chen^1^, Shumei Zou^4^, Haiyan Tian^1^, Ming Wang^1,2^,Lunquan Sun^5^, Zhihui Hao^1^, Yanxin Hu^1*^

^1^ Key Laboratory of Animal Epidemiology of Ministry of Agriculture, College of Veterinary Medicine, China Agricultural University, Beijing, China.

^2^ Key Laboratory of Veterinary Bioproduction and Chemical Medicine of the Ministry of Agriculture, Zhongmu Institutes of China Animal Husbandry Industry Co., Ltd, Beijing, China.

^3^ National Institute for Viral Disease Control and Prevention, Collaboration Innovation Center for Diagnosis and Treatment of Infectious Diseases, Chinese Center for Disease Control and Prevention, Key Laboratory for Medical Virology, National Health and Family Planning Commission, Beijing, China.

^4^ Center for Molecular Medicine, Xiangya Hospital, Central South University, Changsha, China.

**^†^ Contributed to the work equally.**

***** **Corresponding author:**

Dr. Yanxin Hu, Key Laboratory of Animal Epidemiology of Ministry of Agriculture, College of Veterinary Medicine, China Agricultural University, Beijing, P.R.China. Tel: +86 010 62731977. Email: [07033@cau.edu.cn](mailto:07033@cau.edu.cn)

**Table S1. The primer sequences**

|  | **Target name** | **Primers** |
| --- | --- | --- |
| **Influenza A virus** | **MB Tuni-12** | **5’- ACGCGTGATCAGCAAAAGCAGG -3’** |
|  | **MB Tuni-13** | **5’- ACGCGTGATCAGTAGAAACAAGG -3’** |
|  | **PB2** | **5’- AGCGAAAGCAGGTCAATTAT -3’** |
|  |  | **5’- AGTAGAAACAAGGTCGTTTTTAAAC -3’** |
|  | **PB1** | **5’- AGCGAAAGCAGGCAAACCAT -3’** |
|  |  | **5’- AGTAGGAACAAGGCATTTTTTCATG -3’** |
|  | **PA** | **5’- AGCGAAAGCAGGTACTGATCC -3’** |
|  |  | **5’- AGTAGAAACAAGGTACTTTTTTGG -3’** |
|  | **HA** | **5’- AGCAAAAGCAGGGGAA -3’** |
|  |  | **5’- AGTAGAAACAAGGGTGTTTT -3’** |
|  | **NP** | **5’- AGCAAAAGCAGGGTAGATAATC -3’** |
|  |  | **5’- AGTAGAAACAAGGGTATTTTTC -3’** |
|  | **NA** | **5’- AGCGAAAGCAGGGGTTTAAAATG -3’** |
|  |  | **5’- AGTAGAAACAAGGAGTTTTTTGAAC -3’** |
|  | **M** | **5’- AGCGAAAGCAGGTAGATATTG -3’** |
|  |  | **5’- AGTAGAAACAAGGTAGTTTTTTAC-3’** |
|  | **NS** | **5’- AGAAAAAGCAGGGTGACAAA -3’** |
|  |  | **5’- AGTAGAAACAAGGGTGTTTT -3’** |
| **Mouse** | **IFN-β** | **5’- AGATGTCCTCAACTGCTCTC -3’** |
|  |  | **5’- AGATTCACTACCAGTCCCAG -3’** |
|  | **IFN-γ** | **5’- GACTGTGATTGCGGGGTTGT -3’** |
|  |  | **5’- GGCCCGGAGTGTAGACATCT -3’** |
|  | **ISG56** | **5’- GACAAGGCAATCACCCTCTAC -3’** |
|  |  | **5’- GTCTTTCAGCCACTTTCTCCA -3’** |
|  | **AGO2** | **5’- ACATTCCCGCAGGCACAA -3’** |
|  |  | **5’- GTCATCCCAAAGCACGTGGTAG -3’** |
|  | **β-actin** | **5’- GAGACCTTCAACACCCCAGC -3’** |
|  |  | **5’- ATGTCACGCACGATTTCCC -3’** |
| **Human** | **IFN-β** | **5’- ATGACCAACAAGTGTCTCCTCCAAA -3’** |
|  |  | **5’- TTCTTCCAGGACTGTCTTCA -3’** |
|  | **IFN-γ** | **5’- GGCATTTTGAAGAATTGGAAAG -3’** |
|  |  | **5’- TTTGGATGCTCTGGTCATCTT -3’** |
|  | **ISG56** | **5’- GGATTCTGTACAATACACTAGAAACCA -3’** |
|  |  | **5’- CTTTTGGTTACTTTTCCCCTATCC -3’** |
|  | **AGO2** | **5’- GTTTGACGGCAGGAAGAATCT -3’** |
|  |  | **5’- AGGACACCCACTTGATGGACA -3’** |
|  | **GAPDH** | **5’- GCA AATTCCATGGCACCGT -3’** |
|  |  | **5’- TCGCCCCACTTGATTTTGG -3’** |
|  | **IFNAR1** | **5’- TGACGCTGTATGTGAGAA -3’** |
|  |  | **5’- ATAAATGACAAACGGGAG -3’** |
|  | **IFNGR1** | **5’- CTTAGCCTGGTATTCATC -3’** |
|  |  | **5’- CTCTTCACAGACCACCTC -3’** |

**Table S2. The target sequences** **for siRNA transfection**

| **Target name** | **siRNA name** | **sequences** |
| --- | --- | --- |
| **AGO2** | **siAGO2-1** | **5’- GACGGCAGGAAGAAUCUAUTT -3’** |
|  |  | **5’- AUAGAUUCUUCCUGCCGUCTT -3’** |
|  | **siAGO2-2** | **5’- GGGUAAAGUUUACCAAAGATT -3’** |
|  |  | **5’- UCUUUGGUAAACUUUACCCTT -3’** |
|  | **siAGO2-3** | **5’- GCCUGUAUCAAGCUAGAAATT -3’** |
|  |  | **5’- UUUCUAGCUUGAUACAGGCTT -3’** |
| **IFNAR1** | **siIFNAR1-1** | **5’- GCAGCACUACUUACGUCAUTT -3’** |
|  |  | **5’- AUGACGUAAGUAGUGCUGCTT -3’** |
|  | **siIFNAR1-2** | **5’- CCUACUUCCUCCAGUCUUUTT -3’** |
|  |  | **5’- AAAGACUGGAGGAAGUAGGTT -3’** |
|  | **siIFNAR1-3** | **5’-** **GGAAUCCUGGAAACCAUUUTT -3’** |
|  |  | **5’- AAAUGGUUUCCAGGAUUCCTT -3’** |
| **IFNGR1** | **siIFNGR1-1** | **5’- GGAUCUACUUGUGGAUGAUTT -3’** |
|  |  | **5’- AUCAUCCACAAGUAGAUCCTT -3’** |
|  | **siIFNGR1-2** | **5’- GCCUACACCAACUAAUGUUTT -3’** |
|  |  | **5’- AACAUUAGUUGGUGUAGGCTT -3’** |
|  | **siIFNGR1-3** | **5’- CCAGUAUCCUCACUGAAUUTT -3’** |
|  |  | **5’- AAUUCAGUGAGGAUACUGGTT -3’** |


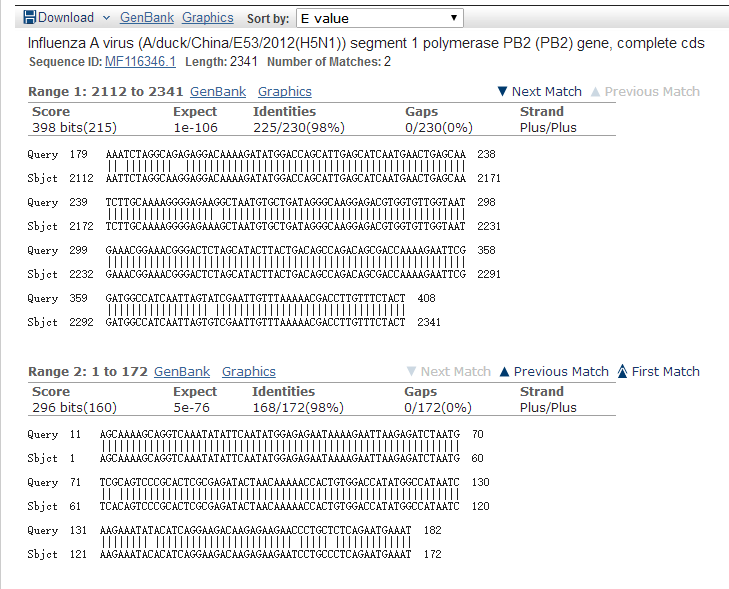


A

B


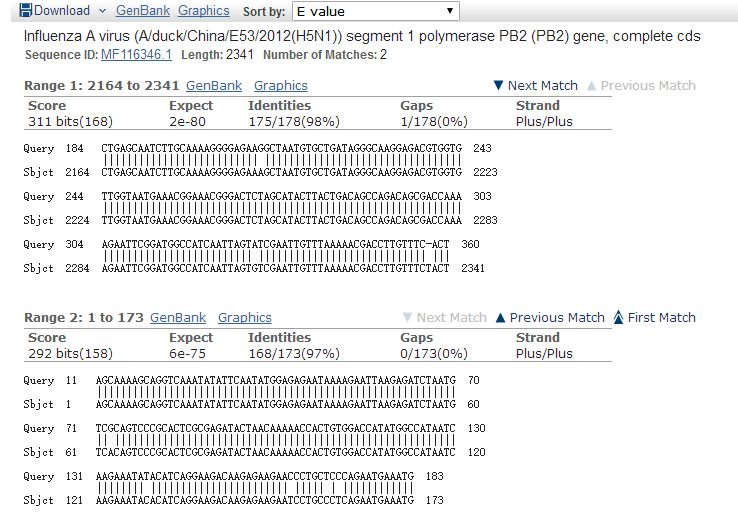


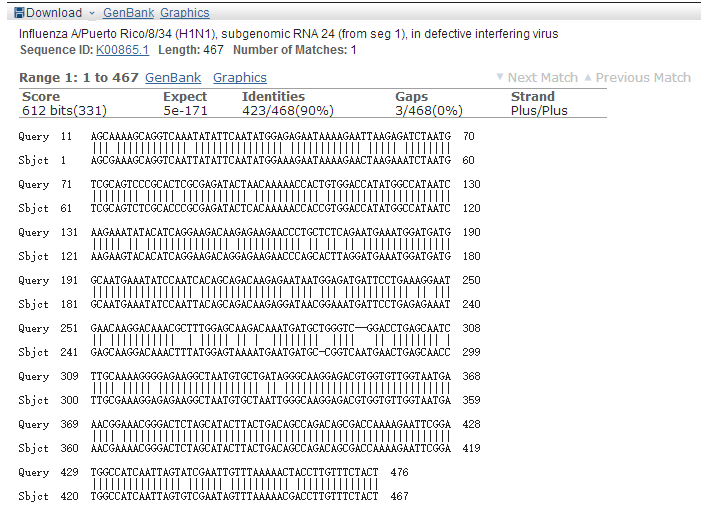


C

D


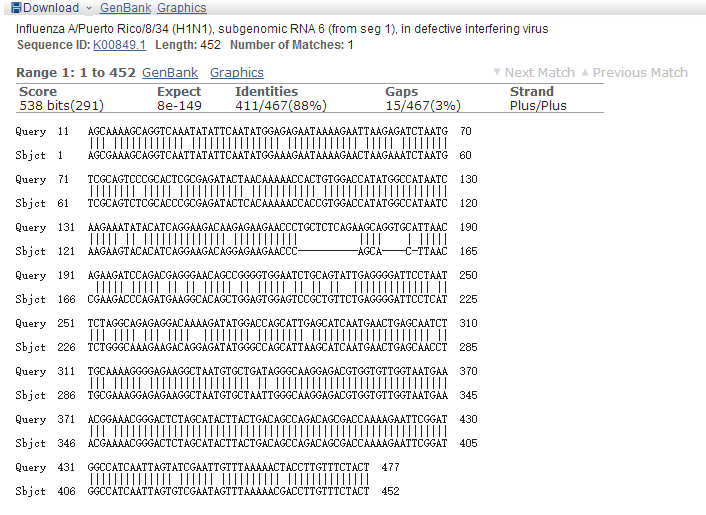


**Fig. S1: Nucleotide sequences of DVGs occured in PB1 gene fragments.** (A-B) Nucleotide sequences of DVGs derived from H5N1-infected HMC-1 cells. (C-D) Nucleotide sequences of DVGs derived from H1N1-infected HMC-1 cells.

**
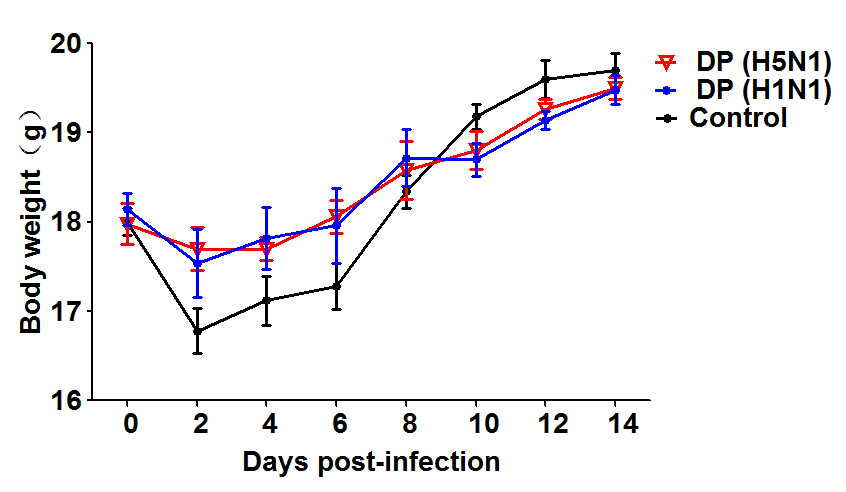
**

**Fig. S2: The infectivity detection of prepared defective particles.** Mice were treated with 25 µl/mouse DP (H5N1), DP (H1N1), or DMEM, respectively. Weight changes were recorded.

**
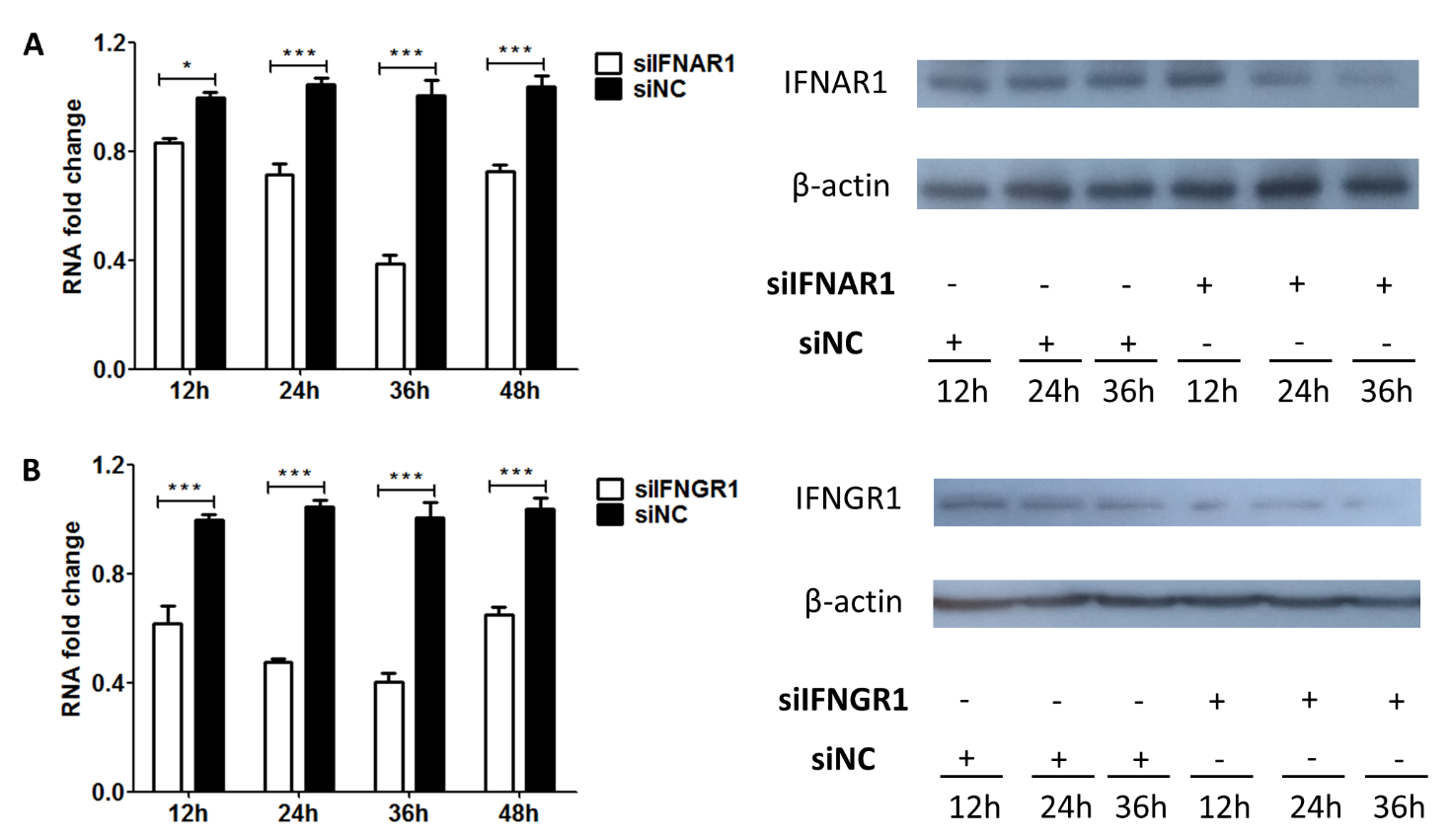
**

**Fig. S3: The detection of transfection efficiency of IFNAR1 and IFNGR1 siRNA.** A549 cells were transfected with siIFNAR1 (A) and siIFNGR1 (B), then the cells and supernatants were collected at the indicated times, respectively. Transfection efficiency of IFNAR1 and IFNGR1 siRNA was assessed using RT-PCR and western blotting, respectively. siNC was a negative control. Graphs are shown from three independent replicates (*P < 0.05; **P < 0.01; ***P < 0.001).
